# Efficient decomposition methods for controlled-*R*_*n*_ using a single ancillary qubit

**DOI:** 10.1038/s41598-018-23764-x

**Published:** 2018-04-03

**Authors:** Taewan Kim, Byung-Soo Choi

**Affiliations:** Electronics and Telecommunications Research Institute, Daejeon, 34129 Korea

## Abstract

We consider decomposition for a controlled-*R*_*n*_ gate with a standard set of universal gates. For this problem, a method exists that uses a single ancillary qubit to reduce the number of gates. In this work, we extend this method to three ends. First, we find a method that can decompose into fewer gates than the best known results in decomposition of controlled-*R*_*n*_. We also confirm that the proposed method reduces the total number of gates of the quantum Fourier transform. Second, we propose another efficient decomposition that can be mapped to a nearest-neighbor architecture with only local CNOT gates. Finally, we find a method that can minimize the depth to 5 gate steps in a nearest-neighbor architecture with only local CNOT gates.

## Introduction

Due to the recent advances in quantum device technology, an arbitrary single-qubit gate or a Z-rotation gate can be implemented with fairly high accuracy, and a small quantum algorithm can be tested. However, even with the gate of a small error rate currently being realized, it is difficult to directly perform scalable quantum computation since it requires that arbitrarily large computations is implemented. In order to overcome this problem, fault-tolerable computation is still needed^1^. Therefore, for reliable quantum computation, all quantum operations of a quantum algorithm should be represented by a universal gate set that arises from a fault-tolerant protocol such as Clifford + *T* gates^2^.

We consider a standard set of universal gates consisting of Hadamard (denoted *H*), phase (*S*), *π*/8 (*T*), and controlled-NOT (CNOT) gates. Although it is known that quantum algorithms have much lower computational complexities than classical algorithms for problem such as factoring large integers^3^, when such quantum algorithm are decomposed into CNOT, *H*, *S*, and *T* gates, the result includes a huge number of gates. Thus, the advantages of quantum computing might be nullified. To enhance the benefits of quantum computation, it is important to use an efficient decomposition of quantum algorithms into universal gates. Here, we first consider the decomposition of single-qubit gates and two-qubit gates. Any single-qubit gate can be decomposed in terms of Hadamard gates and Z-rotation gates *R*_*z*_(*θ*)^4,5^, and there are well-known methods to approximate *R*_*z*_(*θ*) efficiently^6–9^. Next, we consider a controlled*-R*_*n*_ gate as the simplest 2-qubit gate to be decomposed into a universal set of gates. Controlled-*R*_*n*_ gates represent the fundamental part of the quantum Fourier transform (QFT) and many other quantum algorithms. Thus, controlled-*R*_*n*_ decomposition has a significant impact on the overall decomposition of a quantum algorithm. In this work, we propose efficient controlled-*R*_*n*_ decomposition methods as a technique to help enhance the benefits of quantum computation.

## Background

### **Approximation** of *R*_*n*_ gate

An *R*_*n*_ gate is defined as follows:

1 $${R}_{n}=[\begin{array}{ll}1 & 0\\ 0 & {e}^{i\pi {\mathrm{/2}}^{n-1}}\end{array}].$$

The *R*_2_ gate is an *S* gate (or *P* gate), and the *R*_3_ gate is a *T* gate. The *R*_2_ and *R*_3_ gates are included in the universal set. However, *R*_*n*_ for *n* ≥ 4 cannot be exactly decomposed with only a standard set of universal gates^8^. Thus, we should approximate *R*_*n*_ for *n* ≥ 4 to express it with the standard set.

To approximate the *R*_*n*_ gate, we use the gridsynth method^9^. Given a precision *ε* > 0, the approximation of an *R*_*n*_ gate is to find an operator *U* expressible as *H*, *S*, *T* and Pauli operators such that

2 $$||{R}_{n}-U||\le \varepsilon ,$$

where the norm is the operator norm.

The gridsynth algorithm^9^ gives the result of the efficient approximation of an *R*_*n*_ gate in a probabilistic manner. Thus, we estimate the average number of gates for it. From Table 1, we can assume the average numbers of gates for an approximation of an *R*_*n*_ gate as 127, 253 and 379 with *ε* = 10^−5^, 10^−10^, and 10^−15^, respectively. Note that the average number of gates is independent of the rotation angle.

**Table 1:** Average numbers of gates over 10,000 runs for an approximation of *R*_*n*_ with angle *π*/2^*n*−1^.

Angle	Precision 10^−5^	Precision 10^−10^	Precision 10^−15^
*π*/2^3^	126.9226	253.3806	379.3563
*π*/2^4^	126.7122	253.3352	379.4713
*π*/2^5^	126.8313	253.2603	379.0883
*π*/2^6^	126.8625	253.3316	379.3822
*π*/2^7^	126.8923	253.4391	379.0980
*π*/2^8^	126.9019	253.1520	379.9702
*π*/2^9^	126.9230	253.2793	379.0183
*π*/2^10^	126.9107	253.2635	379.2323
*π*/2^11^	126.9982	253.5258	379.3016
*π*/2^12^	126.7677	253.4237	379.1009
*π*/2^13^	126.8485	253.4133	379.3630
*π*/2^14^	126.8366	253.1778	379.3084
*π*/2^15^	126.9337	253.5174	379.2136
Average number of gates	127	253	379

### Zero ancillary qubit method (Method 1)

A controlled-*R*_*n*_ gate is defined as follows:

3 $$\text{Controlled} \mbox{-} {R}_{n}=[\begin{array}{llll}1 & 0 & 0 & 0\\ 0 & 1 & 0 & 0\\ 0 & 0 & 1 & 0\\ 0 & 0 & 0 & {e}^{i\pi {\mathrm{/2}}^{n-1}}\end{array}].$$

**Figure 1:**
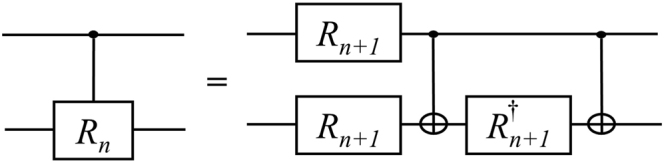
Circuit implementing a controlled-*R*_*n*_ gate with CNOT, *R*_*n* + 1_ and ${R}_{n+1}^{\dagger }$ gates^10^.

Figure 1 shows the circuit of the controlled-*R*_*n*_ gate with 2 CNOTs, 2 *R*_*n*+1_s and 1 ${R}_{n+1}^{\dagger }$ gate. This method is a well-known and fundamental method for the decomposition of a controlled-*R*_*n*_^10^. When we approximate the controlled-*R*_*n*_ with precision 10^−10^, the total number of gates is 761 on average from Table 1. Thus, the approximation of one controlled-*R*_*n*_ requires an excessive number of gates.

### One ancillary qubit method (Method 2)

**Figure 2:**
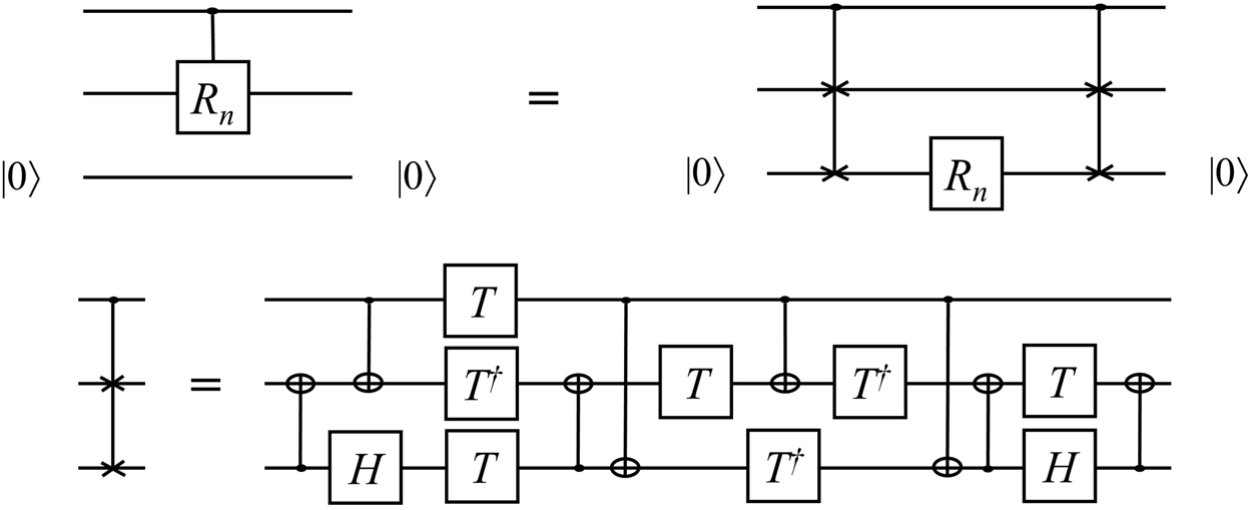
Circuit implementing a controlled-*R*_*n*_ gate with a single ancillary qubit $|0\rangle$^11,12,18^. The ancillary qubit is initialized in and returned to state $|0\rangle$.

Figure 2 shows the circuit of the controlled-*R*_*n*_ gate using a single ancillary qubit. The circuit consists of 1 *R*_*n*_, 16 CNOTs, 4 *H*s, 8 *T*s and 6 *T*^†^s.

As noted in ref.^8^, one advantage of such a circuit is that it reduces the depth with only a small constant overhead. As mentioned earlier, *R*_*n*_ and *R*_*n*+1_ require many gates according to the precision. In the case of the precision 10^−10^, *R*_*n*_ and *R*_*n*+1_ both require approximately 253 gates. Therefore, the approach where a single ancillary qubit is employed appears to be beneficial.

We note that the ref.^11^ offers an approach to implementing a controlled-*U* operation using an ancillary qubit containing an eigenstate of *U*. However, in this paper, we only focus on an approach using $|0\rangle$ state as an ancillary qubit. Thus, we have considered decomposition of controlled-*R*_*n*_ gate in an approach of the ref.^11^. As future work, we will analyze the decomposition of a controlled-*U* operation.

### Controlled-T decomposition based method (Method 3)

The previously known efficient decomposition of a controlled-*T* is shown in ref.^12^. We can observe that the middle *T* gate in ref.^12^ can be replaced with the *R*_*n*_. In this case, controlled-*R*_*n*_ gate can be decomposed into 4 Hadamard gates, 2 Phase gates, 12 CNOT gates, 8 *T* gates, and 1 *R*_*n*_ gate. This result is the best known to date and is the same as in ref.^13^. If we use two ancillary qubits, *T* depth of decompsition of controlled-*T* can be reduced from 5 to 3^13^. However, if we consider only one ancillary qubit, *T*-depth 5 and *T*-count 9 are the best results in decomposition of controlled-*T* gate.

## Results

In this work, we improve the previous method to three ends: to reduce the total number of gates, achieve an efficient layout and achieve a smaller depth.

### Smaller number of total gates (Improvement 1)

We propose an improvement whereby the controlled-*R*_*n*_ consists of a lower total number of gates keeping one *R*_*n*_ gate.

#### **Theorem 1**.

*The controlled-R*_*n*_ *gate can be decomposed with at most one ancillary state* $|0\rangle$ into one *R*_*n*_, eight CNOTs, four *H*s, four *T*s and four *T*^†^s.

**Figure 3:**
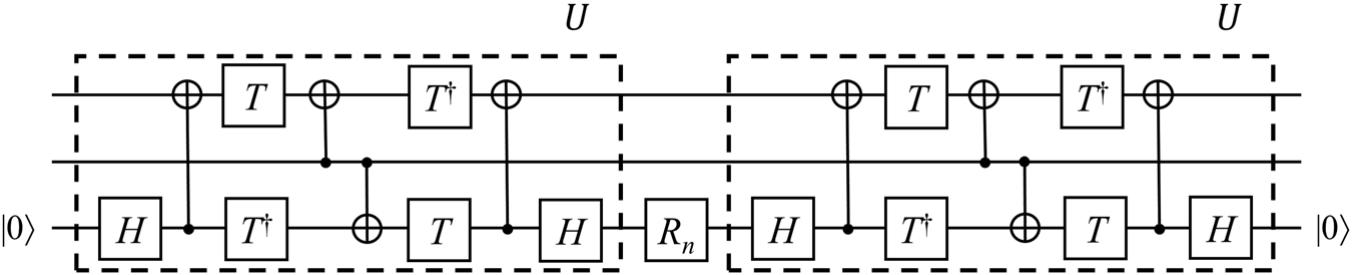
Circuit for the controlled-*R*_*n*_ decomposition for a smaller number of gates.

The proof is given in Section Proofs. The corresponding decomposition is shown in Fig. 3.

The advantage of the proposed method is shown in Table 2. The data were estimated by the ScaffCC program^14^. In particular, in the case of a controlled-*T*, using ancillary qubits results in an exact decomposition of the controlled-*R*_*n*_ and not an approximation. Thus, the gap between Method 1 and Improvement 1 is more larger. The Method 3 is more efficient than the Method 2 in decomposition of controlled-*T*. However, it consist of 12 CNOTs, 4 *H*s, 1 *P*, 1 *P*^†^, 5 *T*s and 4 *T*^†^s. The decomposition includes 27 gates, whereas our decomposition includes only 21 gates. In more detail, *T*-count is the same for ref.^12^ and our method. However, the advantage of our method is reduction by 4 CNOT gates and 2 Phase gates. The reduction of CNOT gates is important since implementation of CNOT gates is physically not easy and controlled-*R*_*n*_ is not the final algorithm^15,16^. Thus, its impact in quantum algorithms will be large. For example, according to module count analysis of ScaffCC Program^14^ for Shor’s algorithm, the controlled-*T* gate is used 641,990,656 times in total. This means that reducing 6 gates in decomposition of the controlled-*T* gate reduces 3,851,943,936 gates in computing of Shor’s algorithm.

**Table 2:** Decomposition of controlled-*T* gate by four methods.

Controlled-*R*_*n*_	Controlled-*T*			
Resource analysis	Method 1	Method 2	Method 3	Improvement 1
Number of qubits (K)	2	3	3	3
Total number of gates	790	35	27	21
Critical path (Q)	528	21	19	17
Reduction rate of total number of gates	1	22.57	29.26	37.62
Reduction rate of KQ	1	16.76	18.53	20.71

Here, the precision for the approximation is 10^−10^, and the reduction rate means the reduction rate for Method 1.

### Efficient layout (Improvement 2)

For practical quantum computing, we should consider the layout of quantum circuits. Since nonlocal two-qubit-gate operation is not allowed in general, a long-range CNOT gate is implemented with several adjacent SWAP gates. In the following theorem, we present an efficient decomposition of a controlled-*R*_*n*_ gate without using nonlocal CNOT gates.

#### **Theorem 2**.

*A controlled-R*_*n*_ *gate can be implemented under the nearest-neighbor-interaction-only architecture with at most one ancillary state* $|0\rangle$ using one *R*_*n*_, twelve adjacent CNOTs, four *H*s, four *T*s and four *T* ^†^s.

**Figure 4:**
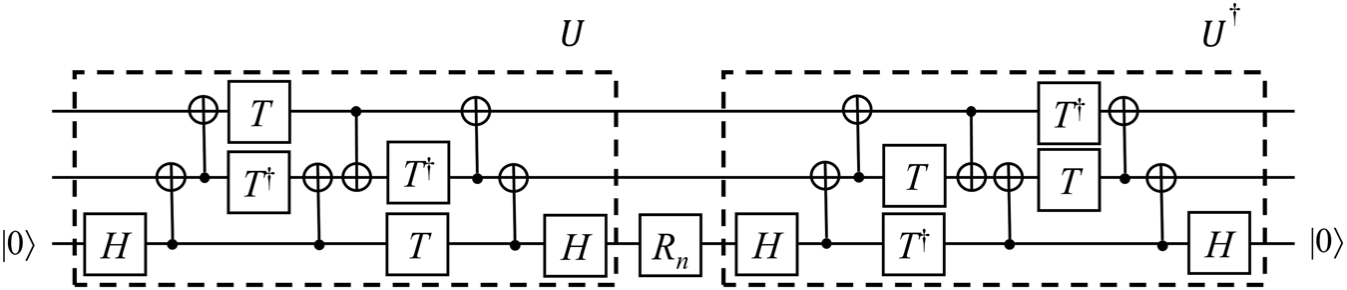
Circuit implementing a controlled-*R*_*n*_ gate for an architecture with only nearest-neighbor interactions.

The proof is given in Section Proofs. The corresponding circuit is shown in Fig. 4. Let us consider one long-range CNOT gate, where the control qubit is the first qubit and the target qubit is the third qubit. Naively, we can decompose such a CNOT gate into one adjacent CNOT gate and two swap gates. The swap gates can be decomposed into three CNOT gates. Thus, the long-range CNOT can be implemented with 7 CNOT gates. More efficiently, the long-range CNOT can be implemented with only 4 CNOT gates^17^. Thus, Method 2 consists of 1 *R*_*n*_, 28 adjacent CNOTs, 4 *H*s, 8 *T*s and 6 *T* ^†^s, while Improvement 2 consists of 1 *R*_*n*_, 12 adjacent CNOTs, 4 *H*s, 4 *T*s and 4 *T* ^†^. Therefore, using our method, we use 16 fewer CNOT gates, 4 fewer *T* gates and 2 fewer *T* ^†^ gates.

### Smaller depth (Improvement 3)

The depth of a circuit means the length of the critical path of the circuit. To ensure an efficient run time of a practical quantum computer, the depth of a circuit should be minimized. For this purpose, we propose a circuit with a smaller depth for a controlled-*R*_*n*_.

#### **Theorem 3**.

*While maintaining the R*_*n*_*-type gate depth 1, the controlled-R*_*n*_ can be implemented with at most one ancillary state $|0\rangle$ with a depth of 5 gates in $\{adjacent\,CNOT,{R}_{n+1},{R}_{n+1}^{\dagger }\}$.

**Figure 5:**
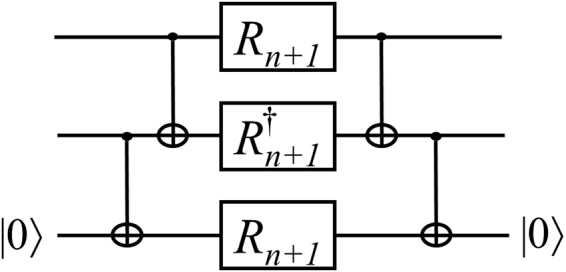
Circuit for the controlled-*R*_*n*_ gate for a smaller depth.

The proof is given in Section Proofs. The corresponding circuit is shown in Fig. 5. Method 2 for the controlled-*R*_*n*_ has a depth of 25, while this circuit only has a depth of 5. Although Method 1 only has a depth of 4, the depth after the approximation of the *R*_*n*_-type gates is nearly twice that of Improvement 3.

We note that from Fig. 8.(a) in ref.^12^, controlled-*S* gate can be decomposed in a depth of 5. However, in the decomposition, two long-range CNOTs is used. Thus, in order to represent controlled-*S* gate only with adjacent CNOTs and *R*_*n*_-type gates, the long-range CNOTs should be transformed into several adjacent CNOTs or layout of qubits should be changed. That is, more resources than in the method of in Fig. 5 are required. According to module count analysis of ScaffCC Program^14^ for Shor algorithm, the controlled-*S* gate is used 641,013,760 times in total. This means that reducing one depth in decomposition of the controlled-*S* gate affects 641,013,760 computing in Shor’s algorithm.

## Efficient decomposition of the quantum Fourier transform

The quantum Fourier transform (QFT) is the key ingredient for quantum factoring and many other quantum algorithms^2^. The total number of gates of the QFT for *n* qubits (denoted *QFT*(*n*)) is obtained as

4 $$QFT(n)=\frac{n(n+\mathrm{1)}}{2}+3\lfloor \frac{n}{2}\rfloor .$$

Now, we compare the total number of gates for the QFT by applying each decomposition method. *QFT*_M1_(*n*), *QFT*_M2_(*n*), *QFT*_M3_(*n*) and *QFT*_I1_(*n*) denote the total number of gates by Method 1, Method 2, Method 3 and Improvement 1, respectively, as follows:

5 $$QF{T}_{{\rm{M1}}}(n)=6n-5+\frac{(n-\mathrm{1)(}n-\mathrm{2)(2}+3c)}{2}+3\lfloor \frac{n}{2}\rfloor $$

6 $$QF{T}_{{\rm{M2}}}(n)=41n-75+\frac{(n-\mathrm{2)(}n-\mathrm{3)(34}+c)}{2}+3\lfloor \frac{n}{2}\rfloor ,$$

7 $$QF{T}_{{\rm{M3}}}(n)=33n-59+\frac{(n-\mathrm{2)(}n-\mathrm{3)(26}+c)}{2}+3\lfloor \frac{n}{2}\rfloor ,$$

8 $$QF{T}_{{\rm{I1}}}(n)=27n-47+\frac{(n-\mathrm{2)(}n-\mathrm{3)(20}+c)}{2}+3\lfloor \frac{n}{2}\rfloor ,$$

where *c* means average number of gates over 10,000 runs for an approximation of *R*_*n*_ with angle *π*/2^*n*−1^ corresponding to the precision of Table 1. For example, if a precision *ε* = 10^−10^ then *c* = 253. Thus, the benefit of Improvement 1 for Method 3 is obtained as

9 $$QF{T}_{{\rm{M3}}}(n)-QF{T}_{{\rm{I1}}}(n)=6n-12+\mathrm{3(}n-\mathrm{2)(}n-\mathrm{3)}=\mathrm{3(}n-\mathrm{1)(}n-\mathrm{2)}$$

for *n*. In this paper, we only consider the error rate in approximation of *R*_*n*_ gate not the overall error rate in approximation of QFT. However, we can notice that Method 3 and Improvement 1 have the same number of *R*_*n*_ gate, and Improvement 1 has smaller number of gates than Method 3. Thus, the overall error rate in approximation of QFT for Improvement 1 might be not greater than that for Method 3. From Table 3 and the above Equations (5–8), it is shown that Improvement 1 is more efficient than Method 1, Method 2 and Method 3.

**Table 3:** Total numbers of gates induced in the approximation for the 3-qubit QFT with precision 10^−5^, 10^−10^ and 10^−15^.

*n*-qubit QFT	*n* = 3			
Precision	Method 1	Method 2	Method 3	Improvement 1
10^−5^ (*c* = 127)	399	51	43	37
10^−10^ (*c* = 253)	777	51	43	37
10^−15^ (*c* = 379)	1155	51	43	37

Note that *c* denotes the expected number of gates obtained in the approximation of the *R*_*n*_ gate.

## Discussion

We have investigated the decomposition problem for the controlled-*R*_*n*_ gate since it is an important two-qubit gate. One method has been proposed that utilized a single ancillary qubit to reduce the number of gates. In this work, we have extended this method for three purposes: to reduce the number of gates, to find a good mapping for an architecture with only nearest-neighbor interactions, and to minimize the critical path. Specifically, we have realized that the proposed method reduces the number of gates for the quantum Fourier transform.

As future work, we will consider three issues. First, we need to check whether the proposed methods are optimal. In addition, it would be interesting to investigate how much performance gain is possible for quantum algorithms such as Shor’s factoring algorithm since it heavily uses the quantum Fourier transform. For more general situations, we need to develop a decomposition method for controlled multi-qubit unitary transforms.

## Proofs

**Proof of Theorem 1**.

### *Proof*.

Let $|\psi \rangle$ be an arbitrary two-qubit state. Then, $|\psi \rangle$ can be represented as

10 $$|\psi \rangle ={\alpha }_{00}|00\rangle +{\alpha }_{01}|01\rangle +{\alpha }_{10}|10\rangle +{\alpha }_{11}|11\rangle ,$$

where *α*_*i*_ are complex numbers and ${\sum }_{i=00}^{11}|{\alpha }_{i}{|}^{2}=1$. Thus,

11 $$\text{Controlled} \mbox{-} {R}_{n}|\psi \rangle ={\alpha }_{00}|00\rangle +{\alpha }_{01}|01\rangle +{\alpha }_{10}|10\rangle +{e}^{i\pi {\mathrm{/2}}^{n-1}}{\alpha }_{11}|11\rangle .$$

Let an unitary operator *U* be an operator denoted by

12 $$U=(I\otimes I\otimes H){C}_{31}({T}^{\dagger }\otimes I\otimes T){C}_{23}{C}_{21}(T\otimes I\otimes {T}^{\dagger }){C}_{31}(I\otimes I\otimes H),$$

where *C*_*ij*_ denotes a CNOT gate with control qubit *i* and target qubit *j*. Then,

13 $$\begin{array}{c}U=|000\rangle \langle 000|+|001\rangle \langle 001|+|010\rangle \langle 010|-|011\rangle \langle 011|\\ \quad \quad +|100\rangle \langle 100|+|101\rangle \langle 101|+i|110\rangle \langle 111|-i|111\rangle \langle 110|.\end{array}$$

Thus,

14 $$\begin{array}{l}U(I\otimes I\otimes {R}_{n})U(|\psi \rangle \otimes |0\rangle )\\ \begin{array}{rcl} & = & U(I\otimes I\otimes {R}_{n})U({\alpha }_{00}|000\rangle +{\alpha }_{01}|010\rangle +{\alpha }_{10}|100\rangle +{\alpha }_{11}|110\rangle )\\  & = & U(I\otimes I\otimes {R}_{n})({\alpha }_{00}|000\rangle +{\alpha }_{01}|010\rangle +{\alpha }_{10}|100\rangle -i{\alpha }_{11}|111\rangle )\\  & = & U({\alpha }_{00}|000\rangle +{\alpha }_{01}|010\rangle +{\alpha }_{10}|100\rangle -i{e}^{i\pi {\mathrm{/2}}^{n-1}}{\alpha }_{11}|111\rangle )\\  & = & {\alpha }_{00}|000\rangle +{\alpha }_{01}|010\rangle +{\alpha }_{10}|100\rangle +{e}^{i\pi {\mathrm{/2}}^{n-1}}{\alpha }_{11}|110\rangle \\  & = & (\text{Controlled} \mbox{-} {R}_{n}\otimes I)(|\psi \rangle \otimes |0\rangle ).\end{array}\end{array}$$

**Proof of Theorem 2**.

### *Proof*.

Let $|\psi \rangle$ be an arbitrary two-qubit state. Then, $|\psi \rangle$ can be represented as

15 $$|\psi \rangle ={\alpha }_{00}|00\rangle +{\alpha }_{01}|01\rangle +{\alpha }_{10}|10\rangle +{\alpha }_{11}|11\rangle ,$$

where *α*_*i*_ are complex numbers and ${\sum }_{i=00}^{11}|{\alpha }_{i}{|}^{2}=1$. Let an unitary operator *U* be the operator denoted by

16 $$U=(I\otimes I\otimes H){C}_{32}{C}_{21}(I\otimes {T}^{\dagger }\otimes T){C}_{12}{C}_{32}(T\otimes {T}^{\dagger }\otimes I){C}_{21}{C}_{32}(I\otimes I\otimes H),$$

where *C*_*ij*_ denotes a CNOT gate with control qubit *i* and target qubit *j*. Then,

17 $$\begin{array}{rcl}U & = & |000\rangle \langle 000|+|001\rangle \langle 001|+|010\rangle \langle 100|+|011\rangle \langle 101|\\  &  & +|100\rangle \langle 010|+|101\rangle \langle 011|-i|110\rangle \langle 111|-i|111\rangle \langle 110|.\end{array}$$

Thus,

18 $$\begin{array}{l}{U}^{\dagger }(I\otimes I\otimes {R}_{n})U(|\psi \rangle \otimes |0\rangle )\\ \begin{array}{rcl} & = & {U}^{\dagger }(I\otimes I\otimes {R}_{n})U({\alpha }_{00}|000\rangle +{\alpha }_{01}|010\rangle +{\alpha }_{10}|100\rangle +{\alpha }_{11}|110\rangle )\\  & = & {U}^{\dagger }(I\otimes I\otimes {R}_{n})({\alpha }_{00}|000\rangle +{\alpha }_{01}|100\rangle +{\alpha }_{10}|010\rangle -i{\alpha }_{11}|111\rangle )\\  & = & {U}^{\dagger }({\alpha }_{00}|000\rangle +{\alpha }_{01}|100\rangle +{\alpha }_{10}|010\rangle -i{e}^{i\pi {\mathrm{/2}}^{n-1}}{\alpha }_{11}|111\rangle )\\  & = & {\alpha }_{00}|000\rangle +{\alpha }_{01}|010\rangle +{\alpha }_{10}|100\rangle +{e}^{i\pi {\mathrm{/2}}^{n-1}}{\alpha }_{11}|110\rangle \\  & = & (\text{Controlled} \mbox{-} {R}_{n}\otimes I)(|\psi \rangle \otimes |0\rangle ).\end{array}\end{array}$$

**Proof of Theorem 3**.

### *Proof*.

Let $|\psi \rangle$ be an arbitrary two-qubit state. Then, $|\psi \rangle$ can be represented as

19 $$|\psi \rangle ={\alpha }_{00}|00\rangle +{\alpha }_{01}|01\rangle +{\alpha }_{10}|10\rangle +{\alpha }_{11}|11\rangle ,$$

where *α*_*i*_ are complex numbers and ${\sum }_{i=00}^{11}|{\alpha }_{i}{|}^{2}=1$. Then,

20 $$\begin{array}{l}{C}_{12}{C}_{23}(|\psi \rangle \otimes |0\rangle )\\ \begin{array}{rcl} & = & {C}_{12}{C}_{23}({\alpha }_{00}|000\rangle +{\alpha }_{01}|010\rangle +{\alpha }_{10}|100\rangle +{\alpha }_{11}|110\rangle )\\  & = & {\alpha }_{00}|000\rangle +{\alpha }_{01}|011\rangle +{\alpha }_{10}|110\rangle +{\alpha }_{11}|101\rangle ,\end{array}\end{array}$$

where *C*_*ij*_ denotes a CNOT gate with control qubit *i* and target qubit *j*.

21 $$\begin{array}{l}({R}_{n+1}\otimes {R}_{n+1}^{\dagger }\otimes {R}_{n+1}){C}_{12}{C}_{23}(|\psi \rangle \otimes |0\rangle )\\ \begin{array}{rcl} & = & {\alpha }_{00}|000\rangle +{\alpha }_{01}|011\rangle +{\alpha }_{10}|110\rangle +{e}^{i\pi {\mathrm{/2}}^{n-1}}{\alpha }_{11}|101\rangle .\end{array}\end{array}$$

Thus,

22 $$\begin{array}{l}{C}_{23}{C}_{12}({R}_{n+1}\otimes {R}_{n+1}^{\dagger }\otimes {R}_{n+1}){C}_{12}{C}_{23}(|\psi \rangle \otimes |0\rangle )\\ \begin{array}{rcl} & = & {\alpha }_{00}|000\rangle +{\alpha }_{01}|010\rangle +{\alpha }_{10}|100\rangle +{e}^{i\pi {\mathrm{/2}}^{n-1}}{\alpha }_{11}|110\rangle \\  & = & (\text{Controlled} \mbox{-} {R}_{n}\otimes I)(|\psi \rangle \otimes |0\rangle ).\end{array}\end{array}$$
